# Genomic Encyclopedia of Bacterial and Archaeal Type Strains, Phase III: the genomes of soil and plant-associated and newly described type strains

**DOI:** 10.1186/s40793-015-0017-x

**Published:** 2015-05-17

**Authors:** William B Whitman, Tanja Woyke, Hans-Peter Klenk, Yuguang Zhou, Timothy G Lilburn, Brian J Beck, Paul De Vos, Peter Vandamme, Jonathan A Eisen, George Garrity, Philip Hugenholtz, Nikos C Kyrpides

**Affiliations:** 1 Department of Microbiology, University of Georgia, Athens, GA, Greece; 2 DOE Joint Genome Institute, Walnut Creek, CA, USA; 3 School of Biology, Newcastle University, Newcastle upon Tyne, UK; 4 China General Microbiological Culture Collection Center (CGMCC), Institute of Microbiology, Chinese Academy of Sciences, Beijing, China; 5 American Type Culture Collection, Manassas, VA, USA; 6 BCCM/LMG Bacteria Collection and Laboratory of Microbiology, Ghent University, Ghent, Belgium; 7 University of California, Davis, CA, USA; 8 Department of Microbiology and Molecular Genetics, Michigan State University, East Lansing, MI, USA, and NamesforLife, LLC, East Lansing, MI, USA; 9 Australian Centre for Ecogenomics, School of Chemistry and Molecular Biosciences, The University of Queensland, Brisbane 4072, QLD, Australia; 10 Current addresses: Microbiologics, St. Cloud, MN, USA; 11 Current addresses: Novozymes North America Inc., Durham 27709, NC, USA

**Keywords:** Genome sequencing, Type stains, Prokaryotes

## Abstract

The Genomic Encyclopedia of *Bacteria* and *Archaea* (GEBA) project was launched by the JGI in 2007 as a pilot project to sequence about 250 bacterial and archaeal genomes of elevated phylogenetic diversity. Herein, we propose to extend this approach to type strains of prokaryotes associated with soil or plants and their close relatives as well as type strains from newly described species. Understanding the microbiology of soil and plants is critical to many DOE mission areas, such as biofuel production from biomass, biogeochemistry, and carbon cycling. We are also targeting type strains of novel species while they are being described. Since 2006, about 630 new species have been described per year, many of which are closely aligned to DOE areas of interest in soil, agriculture, degradation of pollutants, biofuel production, biogeochemical transformation, and biodiversity.

## Background

The Genomic Encyclopedia of Bacteria and Archaea was launched in 2007 with the aim of sequencing 250 type strains from branches of the tree of life with low sequence representation [1]. The GEBA pilot project, which encompassed the first 56 genomes, provided convincing evidence of the value of a phylogeny-driven selection of target strains for the discovery of new protein families and enhancing the accuracy of sequence binning methods commonly used in metagenome projects [1]-[4]. In 2012, GEBA was extended with the project entitled *Genomic Encyclopedia of Type Strains, Phase I: the one thousand microbial genomes* project [5]. Major goals were (i) to demonstrate the value of phylogenetic diversity as a primary criterion for generating genome sequences, (ii) to develop the necessary framework, technology and organization for large-scale sequencing of microbial genomes, and (iii) to cover as much as possible of the genomic diversity in the microbial part of the tree of life. In 2013, a second project entitled *Genomic Encyclopedia of Archaeal and Bacterial Type Strains, Phase II: from individual species to whole genera*, targeted another 1000 genome sequences and strain selection shifted to complete clusters of all the type strains in selected genera and small families. This approach was enabled by the pipeline developed in KMG-I to automate most steps from sequencing, to annotation, and data deposition. We note that there has also been an associated “microbial dark matter” project at DOE-JGI to sequence the genomes of phylogenetically novel, uncultured taxa using a single cell whole genome amplification strategy (see[6]) First results from that project have been published [7], and it too has moved into its own advanced phase. This project is not discussed further since our focus is on cultured organisms.

With the completion of KMG-I expected in the near future, targets have now been selected for KMG-II sequencing. The progress of this effort can be monitored at the Microbial Earth Project [8]. As of December 2014, draft or complete genome sequences were available for 1,763 of the 12,239 type strains described (Garrity and Parker, personal communication). In addition, according to GOLD sequencing projects were underway for 1,610 strains [9], leaving 8,866 additional type strains without plans for sequencing in the immediate future. Anticipating the completion of KMG-II within the next year, a Phase III GEBA project or KMG-III is proposed here to maintain the efficiencies of scale and provide a continuous source of DNA for sequencing of the genomes of microbial type strains.

What are type strains of species and why do we want to sequence them? By definition, type strains are descendants of the original isolates that were the basis for species descriptions, as defined by the Bacteriological Code, and exhibit all of the relevant phenotypic and genotypic properties cited in the original published taxonomic circumscriptions [10]. However, their importance in nomenclature is only one reason for sequencing. By the principles of nomenclature, a type strain cannot be identical with any other type strain. Since 1987, the difference between type strains has generally been defined in genetic terms of <70% DNA:DNA hybridization under optimal conditions and a change in the melting temperature of hybrid DNAs (ΔTm) of >5 °C [11]. In terms of genomic sequences, this level of diversity is equivalent to about 69% conserved DNA (or 85% conserved genes) and 95% average nucleotide identity (ANI) among this conserved DNA [12],[13]. In terms of phenotypic similarity, species generally possess similarity values as defined by numerical taxonomy of >70%, which is close to the limit of significance [14]. Thus, any two properly described type strains must be substantially different. For context, if these same criteria were applied to mammals most primates would be members of the same species [15]. Of additional importance, virtually all type strains are available in pure culture (except in the cases of some symbionts and other non-cultivable species defined prior to the 2001 revisions to the Bacteriological Code). The implication is that genomic sequences of type strains will be based upon well-documented biological specimens that will remain available for further study for the foreseeable future. Thus, it will be possible to experimentally verify the sequence should ambiguities or errors subsequently be identified, complete draft sequences if new methodologies are developed, collect additional types of information about the strains, and experimentally test hypotheses derived from the genome sequences.

## The importance of this research

There are a number of very different but equally valid reasons to sequence the genomes of type strains.

1) Currently, genomic sequences of cultured organisms have sampled about 3.6 % of the estimated phylogenetic diversity of the prokaryotes on earth. If the genomes of all the known type strains were sequenced, this sampling is estimated to increase to about 15% [7]. Given the enormous diversity of prokaryotes, this is not an insignificant fraction.

2) Genomic sequences of type strains will complement metagenomic and metatranscriptomic sequencing efforts, which commonly yield large numbers of short sequences from complex environmental communities. The genomic sequences of type strains will facilitate the identification of gene fragments and provide whole-genome contexts for individual genes.

3) The phenotype and other physiological properties of type strains are frequently well characterized. Genomic sequencing of type strains will enable analyses associating gene content with function, providing an opportunity to validate genome-based metabolic reconstructions.

4) Most type strains were described due to their environmental, medical or commercial importance. The genomic sequence will provide insights into the metabolism, stress responses, adaptations to commensalism, evolution and other processes important to the role of the microorganism. Thus, it contributes greatly to our knowledge of the processes in which these prokaryotes play fundamental roles.

5) Identification of prokaryotes is still a major challenge that hinders many practical applications. Genomic sequencing of type strains will provide the tools that greatly facilitate identification.

6) The genomes of the type strains will provide the high resolution data necessary to resolve the numerous phylogenetic ambiguities limiting prokaryotic taxonomy.

Lastly, we view this effort as transitional by providing the support needed to make genome sequencing technology widely available. As more genomic sequences enter the public databases, their value will become more apparent to most investigators and editors of major journals. Given the anticipated further rapid decreases in cost of sequencing, we expect that genomic sequences will become a routine component of the description of new type strains. Thus, our goal is to place this technology within the reach of a large group of investigators until it becomes a widely accepted component of the description of novel species. We anticipate that once large sequencing projects such as GEBA have sequenced a significant proportion of the type strains, community participation will insure that the database of type strain genomic sequences will remain current. Genomic sequencing is the natural successor of 16S rRNA sequencing, which was introduced in the description of type strains over two decades ago and has now become routine.

## Project design

This project focuses on type strains of prokaryotes associated with soil or plants and their close relatives as well as type strains from newly described species. Even in the age of microchips and Martian landers, soil is the foundation of civilization. It is the basis for agriculture and ultimately the source of most food, timber, and other bioproducts. It contains the largest microbial community outside our own microbiome with which we are in daily contact [15],[16]. The genomic sequences of prokaryotes from soil will provide insights into soil fertility, nutrient cycling, and soil biochemistry. We will also learn more about how soils mineralize pesticides, produce greenhouse gases such as nitrous oxide, or consume greenhouse gases like methane. Likewise, plant biomass is the largest reservoir of living material in the biosphere, far exceeding the amounts in animals and, very likely, microorganisms [17]. Understanding the microbiology of soil and plants is critical to many areas, such as biofuel production from biomass, biogeochemistry, and carbon cycling.

**Figure 1:**
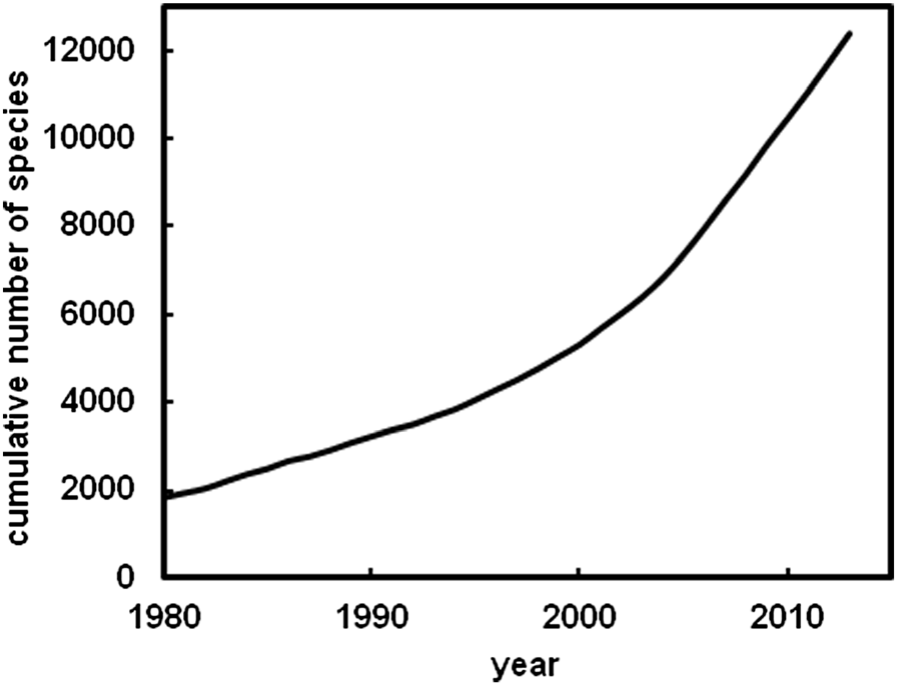
Cumulative number of prokaryotic species described since publication of the Approved List in 1980 and 2013. Modified from Euzéby and Parte [List of Prokaryotic Names with Standing in Nomenclature: http://www.bacterio.cict.fr/].

We are also proposing the sequencing of type strains of novel species while they are being described. Since 2006, investigators from around the world have validly described about 630 new species per year (Figure 1). Many of these species are closely aligned to areas of great general interest. An informal survey of the sources of these new species indicates that about 35% are from soil or plant-associated. Another 55% are from other free-living sources, such as seawater, sediments and the deep subsurface. Many of these include species involved in degradation of pollutants, biofuel production, biogeochemical transformations, and biodiversity.

With some notable exceptions, genomic sequences are not part of the descriptions for most of these species. However, there would be significant value if this were to change. Therefore, we propose that the genomes of novel prokaryotes would be sequenced when culture collections issue the Certification of Availability, a step required for the description of all novel species. This certificate ensures that the culture is available without restriction for other investigators. In addition, the collections routinely ensure that the culture is pure and possesses properties consistent with those claimed by the investigator. Because the original investigator will provide the DNA, access to DNA sequences from type strains will greatly increase.

## Selection of target organisms

We expect that 60% of the target type strains will be selected based on their isolation from soil or plant-associated material. Closely related strains from the same genus or family from other non-human sources will also be included to facilitate comparative analyses. Type strains from human sources are excluded to avoid overlap with existing human microbiome projects. However, it is anticipated that these type strains will be sequenced by other projects and be available for comparative analyses in the near future. The DNA for the KMG III strains will be mostly provided by the culture collections working on the project. The remaining 40% will be type strains from newly described species. The DNA for these genomes will be solicited directly from investigators working on these organisms (see below). We will request proposals for these genome sequencing projects that will include 1) a justification of how the project is consistent with the mission of the KMG III and 2) a copy of the Certificate of Availability, which demonstrates that the culture has been deposited in an internationally recognized culture collection. Upon approval by a committee of the coauthors, the individual investigators working with these strains will be responsible for providing the DNA and metadata associated with that organism.

## Organism growth and nucleic acid isolation

The culture collections at DSMZ and the ATCC are partners in GEBA Phase II, and the work proposed here will be performed in close collaboration with these collections. They will be joined by the China General Microbiological Culture Collection Center (CGMCC), which possesses a large collection of type strains isolated in China, and the Belgian Coordinated Collections of Microorganisms (BCCM), which has great expertise in the type strains of plant-associated bacteria. Because preparation of DNA is now the limiting factor in genome sequencing and many fastidious organism are not easily grown by culture collections, individual investigators will be encourage to submit DNA from laboratory collections of suitable quality. QC procedures will also be standardized for individual investigators, and all DNAs will go through a careful QC procedure before being shipped to the JGI for sequencing.

**Figure 2:**
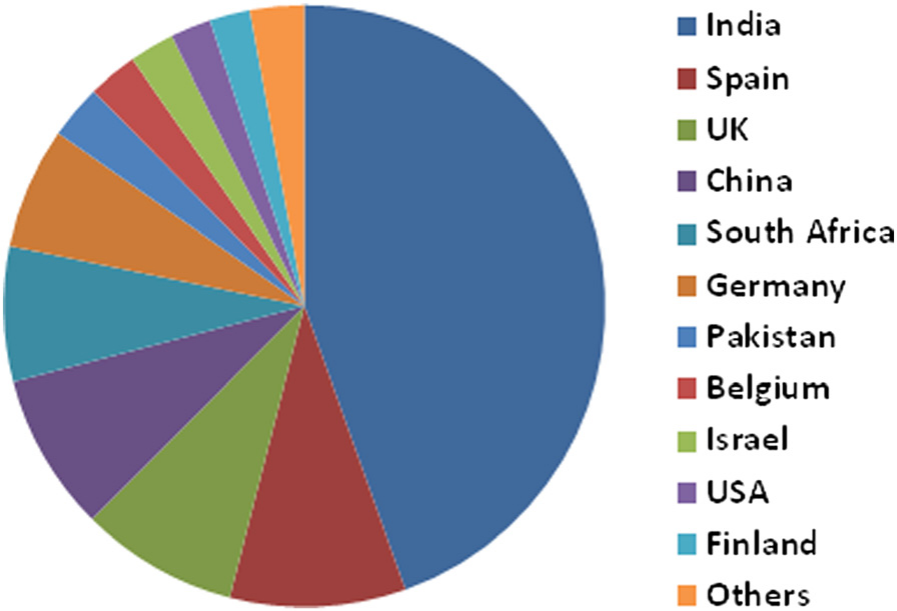
Nationalities of the contributors to the GEBA phase II and III sequencing proposals for the KMG projects. Others include Italy, Mexico and Portugal.

**Figure 3:**
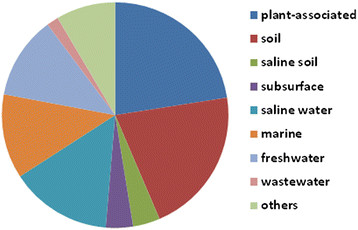
Sources of the type strains contributed to the KMG projects by individual investigators. Others include air, sludge, hot springs, wall painting, and unknown.

A test of the interest by individual investigators was performed in the spring of 2014. Requests for DNA were sent to editors of the leading systematics journals and members of Bergeys International Society for Microbial Systematics. Proposals to sequence genomes of 589 type strains were received as of Dec. 1, 2014. Of these, 413 proposals were approved for either GEBA phase II or phase III sequencing projects. The major reason strains were not approved was that the sequence was either completed or in progress elsewhere. Proposals were approved from 14 countries, which is indicative of large international participation (Figure 2). About half of the type strains were plant-associated or from soil or saline soil, habitats targeted in KMG III (Figure 3). Two physiological groups were also well represented in this collection. Strains from saline environments and phototrophs represented 18 and 20 % of the type strains, respectively. These groups are of special interest because of their adaptations to extreme environments and roles in CO_2_ fixation.

## Sequencing approach

The major sequencing technology developments over the last years renders a project of this scale not only technically but also economically feasible. The bulk of the sequencing will be completed using high throughput sequencing on the HiSeq Illumina platform. For a subset of the target strains, Pacific Biosciences single molecule real time sequencing technology will be used, enabling the generation of complete or near complete genome sequences.

### The size and nature of the larger user community

As with the other GEBA projects, data will be immediately released to the public through a variety of channels [18],[5]. No restrictions will be associated or imposed with the data release, thus allowing the community to make immediate and full use of our findings. The grand scale of the project and the lack of focus on specific applications will most certainly generate novel information of broad scientific interest and benefits for the community at large. Nevertheless, we envision four major stakeholders within the scientific community with vital interests in the genomic sequences of type strains covered in this proposal. **First**, agricultural researchers will be able to use these genomes to better understand plant diseases, the transfer of nutrients to and from the plant in the rhizosphere, and plant symbioses with N_2_-fixing and other prokaryotes. **Second**, for environmental scientists working in soil and plant systems, these type strains will provide crucial information for interpreting metagenomic and metatranscriptomic data sets encompassing complex soil and root-associated microbial communities. **Third**, for bioinformaticians interested in genome structure-function relationships, the availability of large data sets of genomic sequences from phenotypically well characterized strains will provide new opportunities for correlating phenotypic traits with genomic sequences and other functional studies. **Lastly**, as more genomes become available for specific groups, the applications of genome-based systematics will revolutionize the classification of prokaryotes [19],[20]. Sequences based tools for the definition of species such as ANI or Genome-to-Genome-Distance values [13],[21],[22] will replace the imprecise and error prone wet lab determinations of DNA-DNA hybridizations. By providing more reliable and complete data, it will also allow formation of more accurate groupings of higher taxa. In addition, it will provide new insights into the evolution of these prokaryotic groups through both vertical and horizontal mechanisms of gene transfer.

The selection of soil and plant-associated type strains for sequencing will provide strong support for DOE missions in alternative energy production, global carbon sequestration, and biogeochemistry. Alternative energy production, especially biofuel production from biomass, relies heavily on improved and more efficient agriculture to produce inexpensive biomass. Genomic sequencing will contribute to alternative energy production by furthering our understanding of soil fertility, biotransformations of herbicides and pesticides, bacterial diseases of plants, and practical questions in crop management. Similarly, genomic sequencing of plant-associated prokaryotes will provide insights into basic questions in plant-prokaryotic symbioses as well as potential applications in N_2_-fixation and nutrient assimilation. Soil and plants contain 1500 Pg and 560 Pg of carbon, respectively, compared to 750 Pg of carbon in the earth’s atmosphere (1 Pg = 10^15^ g). Given the enormity of these reservoirs, they are important sources and sinks of inorganic and organic carbon.

Genomic sequencing of type strains from soil will provide insights into many of the carbon fluxes from soil. Of special interest are processes and enzymes for degradation of recalcitrant organic compounds, methane-oxidizing bacteria, and CO_2_-fixation among soil autotrophs such as nitrifiers. Because soil is a sink for atmospheric CO_2_ and CH_4_ as well as a source for the greenhouse gas N_2_O, insight into these processes is expected as well. Genomic sequencing of plant-associated type strains will provide insights into processes such as sequestration of newly fixed plant organic carbon by soil microorganisms and mineralization or degradation of complex plant polymers. Finally, soil is a major repository for toxic metals, radionucleotides and organic contaminants. Genomic sequencing of type strains from soil will provide insight into the microbial responses to these compounds, better informing the decision-making process for environmental remediation and providing a mechanistic understanding of bioremediation.

## Conclusion

Phase III of the GEBA project demonstrates that it is possible to obtain significant assistance in the genome sequencing of type strains from the international community of bacteriologists. Experts in individual taxa are willing to provide DNA from the type strains in their laboratory collections for sequencing at JGI. The genome sequences provide these experts great insights into questions specific to their organisms as well as those of interest to the broader community of prokaryotic biologists.

## Abbreviations

ATCC: American Type Culture Collection

BCCM: Belgian Coordinated Collections of Microorganisms

CGMCC: China General Microbiological Culture Collection Center

DSMZ: Deutsche Sammlung von Mikroorganismen und Zellkulturen

GEBA: Genomic Encyclopedia of *Bacteria* and *Archaea*

JGI: Joint Genome Institute

KMG: Thousand Microbial Genomes

## Competing interests

The author(s) declare that they have no competing interests.

## Author’s information

Not applicable.

## Authors’ contributions

The manuscript was written largely by WBW from earlier drafts by TW, HPK and NK. YZ, TGL, BJB, PDV, PV, JAE, GG, and PH provided additional important intellectual content. All authors read and approved the final manuscript.
